# A mixed methods study assessing the adoption potential of a saliva-based malaria rapid test in the Democratic Republic of Congo

**DOI:** 10.1186/s12936-023-04599-y

**Published:** 2023-06-08

**Authors:** John Diaz, Cody Gusto, Kaci McCoy, Colby Silvert, Joseph A. Bala, Joseph Atibu, Antoinette Tshefu, Melchior Mwandagalirwa, Rhoel R. Dinglasan

**Affiliations:** 1 Gulf Coast Research and Education Center, University of Florida, 1200 N. Park Road, Plant City, FL 33563 USA; 2 Department of Agricultural Education and Communication, University of Florida, Rolfs Hall, Gainesville, FL 32611 USA; 3 Department of Infectious Diseases and Immunology, Emerging Pathogens Institute, College of Veterinary Medicine, University of Florida, 2055 Mowry Road, Rm 375, Gainesville, FL 32611 USA; 4 Kinshasa School of Public Health, Kinshasa, Democratic Republic of the Congo

**Keywords:** Adoption, Malaria, Rapid diagnostic test, Saliva-based, Subclinical infection, Treatment

## Abstract

**Background:**

The reliance on blood for thin and thick blood smear microscopy—using a relatively invasive procedure has presented challenges to the use of reliable diagnostic tests in non-clinical settings at the point-of-need (PON). To improve the capacity of non-blood-based rapid diagnostic tests to confirm subclinical infections, and thereby identify and quantify the human reservoir at the PON, a cross-sectoral collaboration between university researchers and commercial partners produced an innovative, non-invasive saliva-based RDT capable of identifying novel, non-hrp2/3 parasite biomarkers. While this new saliva-based malaria asymptomatic and asexual rapid test (SMAART-1) shows increased detection sensitivity and precision potential by identifying a new *P. falciparum* protein marker (PSSP17), appraising its utility in the field—particularly with respect to its adoption potential with children and adults in high risk, endemic regions—is necessary to warrant its continued development.

**Methods:**

The purpose of this study was to assess the acceptability and adoption potential of the SMAART-1 at select PON sites in the Kinshasa Province. Teachers, community health workers, nurses, and laboratory technicians participated in data collection at three distinct community sites in Kinshasa Province, Democratic Republic of the Congo. Three data collection methods were utilized in this mixed methods study to provide an overarching acceptability evaluation of the SMAART-1 at PON field sites: observation checklists of SMAART-1 implementation, focus group discussions, and surveys with local health care practitioners—particularly teachers and community health workers.

**Results:**

Findings indicate participants were interested in and supportive of the SMAART-1 protocol, with approximately 99% of the participants surveyed indicating that they either “agreed” or “strongly agreed” with the statement that they “would use the saliva-based malaria asymptomatic rapid test as part of a community malaria detection and treatment programme.” Data also suggest that the protocol was broadly appealing for its testing sensitivity and ease of use.

**Conclusions:**

The SMAART-1 protocol’s clinically reliable results demonstrate a promising new level of sensitivity and precision for detecting parasite biomarkers. This study’s mixed-methods assessment of the protocol’s utility and adoption potential in the field, with a target user audience, advances its development and points to opportunities to formalize and expand evaluation efforts.

## Background

Malaria is an acute febrile disease principally caused by *Plasmodium* parasite species; the parasite is transmitted in endemic countries through the bite of infected female *Anopheles* mosquitoes. Despite substantial progress in malaria control due to advances in prevention and treatment interventions such as insecticide-treated nets (ITNs), indoor residual spraying (IRS), preventative chemotherapies, and anti-malarial vaccines [1], malaria remains one of the most devastating infectious diseases, with substantial public health and socio-economic impacts for malaria endemic countries [2]. According to the World Health Organization (WHO), there was an estimated 241 million malaria cases across 85 endemic countries in 2020—an increase from 227 million estimates cases in 2019 [3]. An estimated 627,000 deaths were attributed to malaria in 2020, an increase of approximately 70,000 deaths over the previous year [3]. The WHO notes the increase in the rate of deaths was largely attributable to substantial disruptions of both prevention and treatment services due to COVID-19 and a methodological change in how the agency calculated malaria mortality in 32 sub-Saharan African nations that account for roughly 93% of malaria deaths globally [3]. The new cause-of-death assessment method revealed that malaria had an even greater impact on child mortality in Africa and confirmed that four African nations carried the majority of malaria disease burden, with Nigeria (31.9%), the Democratic Republic of Congo (13.2%), Tanzania (4.1%), and Mozambique (3.8%) accounting for over half of all malaria deaths worldwide [3].

Despite the concerning COVID-related service disruptions addressed above, the documented persistence if not resurgence of malaria suggests a waning efficacy of critical prevention, diagnostic, and treatment interventions. These impacts also call into question the achievability of the malaria mitigation targets established in the WHO’s Global technical strategy for malaria 2016–2030 framework (GTS), such as reducing both malaria case incidence and mortality by at least 90% by 2030 [4]. One avenue to increase the likelihood of achieving these ambitious goals is to improve the accessibilityand accuracy of diagnostic testing worldwide [4]. This requires comprehensively appraising the efficacy of established malaria detection strategies, as well as supporting the development of emergent diagnostic tools [5]. While thin and thick blood smear microscopy remains a reliable, quality-assured standard for confirming malaria infection, the technique requires a sufficient level of expertise and training to execute, as well as equipment and that may be cost-prohibitive for many rural health facilities in malaria-endemic regions [5, 6]. Rapid diagnostic tests (RDTs), by contrast, afford health practitioners an opportunity to identify the presence of malaria antigens with a scaleable, deployable, low-cost technique that provides results quickly as RDTs do not require highly-skilled labour, electricity, or specialized equipment [5, 7]. Malaria RDTs can, however, be unreliable in detecting low levels of malaria parasites in a sample and may frequently produce both false negative and false positive results [5, 8]. Additionally, *Plasmodium falciparum* mutant populations have emerged around the globe, which no longer express the histidine-rich protein 2 (*hrp2*) biomarker for current blood-based RDTs, and subsequently go undetected by the majority of RDTs [9, 10].

Both microscopy and blood-based RDTs are limited in their capacity to detect extremely low-density subclinical (i.e., asymptomatic) infections of *P. falciparum*, which have been identified as concerning portions of ongoing malaria transmission and potential factors in the stalling of malaria elimination progress over recent years [9–12]. The reliance on blood, a relatively invasive procedure, also presents a challenge to their use in non-clinical settings at the Point-of-Need (PON). To improve the capacity of diagnostic tests to confirm subclinical infections and thereby identify and quantify the human reservoir at the PON, a cross-sectoral collaboration between researchers from the University of Florida’s Emerging Pathogens Institute and commercial partners produced an innovative, non-invasive saliva-based RDT capable of identifying novel, non-*hrp2/3* parasite bio-markers [12]. While this new saliva-based malaria asymptomatic and asexual rapid test (SMAART-1) shows increased detection sensitivity and precision potential by identifying a new *P. falciparum* protein marker (PSSP17), appraising its acceptability to end-users in the field—particularly with respect to its adoption potential with children and adults in regions with high endemicity—is necessary to warrant its continued commercial development. Although, SMAART-1 is currently in commercial development, and gauging acceptability and stakeholder input presently informs the refinement of the commercial product [13].

The purpose of this study was therefore to assess the acceptability and adoption potential of the SMAART-1 at three PON sites in the Kinshasa Province of the Democratic Republic of the Congo (DRC). By leveraging field data (observation checklists of SMAART-1 implementation, focus groups, and surveys) collected from all individuals involved in malaria surveillance and control in the DRC, including National Malaria Control Programme (NMCP)-trained teachers and health professionals, such as community health workers (CHWs), nurses, and laboratory technicians, the implementation and adoption potential of the SMAART-1 was assessed as a commercially viable RDT alternative to prevailing diagnostic tests that currently struggle to reliably detect subclinical malaria infection.

### Theoretical framework

Tenants of Rogers’ (2003) Diffusion of Innovation (DOI) theory were leveraged to apply evaluative benchmarks for the adoption potential of SMAART-1 relative to established diagnostic tests. The DOI has long been applied across sectors to both explain and predict how certain innovations (i.e., ideas, technologies, or behavioral practices) “diffuse” (i.e., spread) throughout a population over time [14, 15]. Conventionally used to retrospectively dissect the successes and failures of an innovation launch, more recent studies have applied the DOI to predict how the model may “…accelerate the pace of adoption, increase the number of adoptions, enhance the quality of innovation implementation, sustain the use of worthy innovations, and, as ultimate outcomes, demonstrate innovation effectiveness at individual client and client system levels” [16]. Central to the DOI is the assumption that the novelty of a given innovation generates uncertainty for a targeted audience, and that diffusion of the innovation is primarily a result of reducing that uncertainty [14, 15].

The DOI advances five attributes that can be used to better understand how a population perceives the favourability of an innovation, under the assumption that successfully meeting an audience’s expectations for these conditions results in the innovation’s rapid adoption: relative advantage, compatibility, complexity, observability, and trialability [14]. Relative advantage is the degree to which the innovation under consideration is perceived to be “better” to a prevailing idea, technology, or behaviour, with respect to perceived economic advantage, convenience, overall satisfaction, or whatever conditions a user may consider [14]. In the context of this study, relative advantage was applied to measure the extent to which study participants (i.e., teachers and health professionals) perceived the SMAART-1 as being advantageous over prevailing diagnostic tests—particularly blood-based RDTs. Compatibility is a measure of the extent to which a potential adopter believes an innovation is well-suited to their own values, past experiences, habits, or perceived needs [14]. In this study, compatibility served to assess the degree to which the SMAART-1 was compatible with the values and norms of teachers and health professionals. Complexity is the degree to which a potential adopter believes an innovation is difficult to understand, implement, or use [14]. Complexity was applied in this study to measure the extent to which participants in this study perceived the SMAART-1 innovation to be difficult to adopt and administer to their patients. Observability is a measure of the extent to which a potential adopter believes the results of the innovation are visible or perceptibly apparent [14]. Though not specifically targeted in this study, observability in this context would be operationalized as a measure of how easily participants believed they could obtain and interpret SMART-1 test results. Finally, trialability is the degree to which a potential adopter believes an innovation can be trialed, experimented with, or temporarily implemented prior to full adoption [14]. Trialability was not directly operationalized in this study, but could be applied to measure the extent to which teachers, health professionals, and other practitioners felt they could trial SMAART-1 protocols prior to adopting the test into their respective health service operations. Logistical and respondent burden (e.g., survey fatigue and time availability) considerations discouraged the application of a survey instrument that fully incorporated all five of the DOI attributes. Thus, in consultation with local partners and enumerators, a modified, abbreviated DOI index was applied which concentrated on the relative advantage, compatibility, and complexity attributes of the SMAART-1 innovation.

## Methods

Three data collection methods were utilized in this study to provide an overarching acceptability evaluation of the SMAART-1 at PON field sites: observation checklist assessments, surveys, and focus group discussions (FGDs). This study, therefore, applied a convergent mixed methods design in which multiple forms of data—pertaining to the same topic—are collected simultaneously and then are analysed by integrating, comparing, and contrasting common and unique findings from the different sources [17].

### Study location and participant overview

**Fig. 1:**
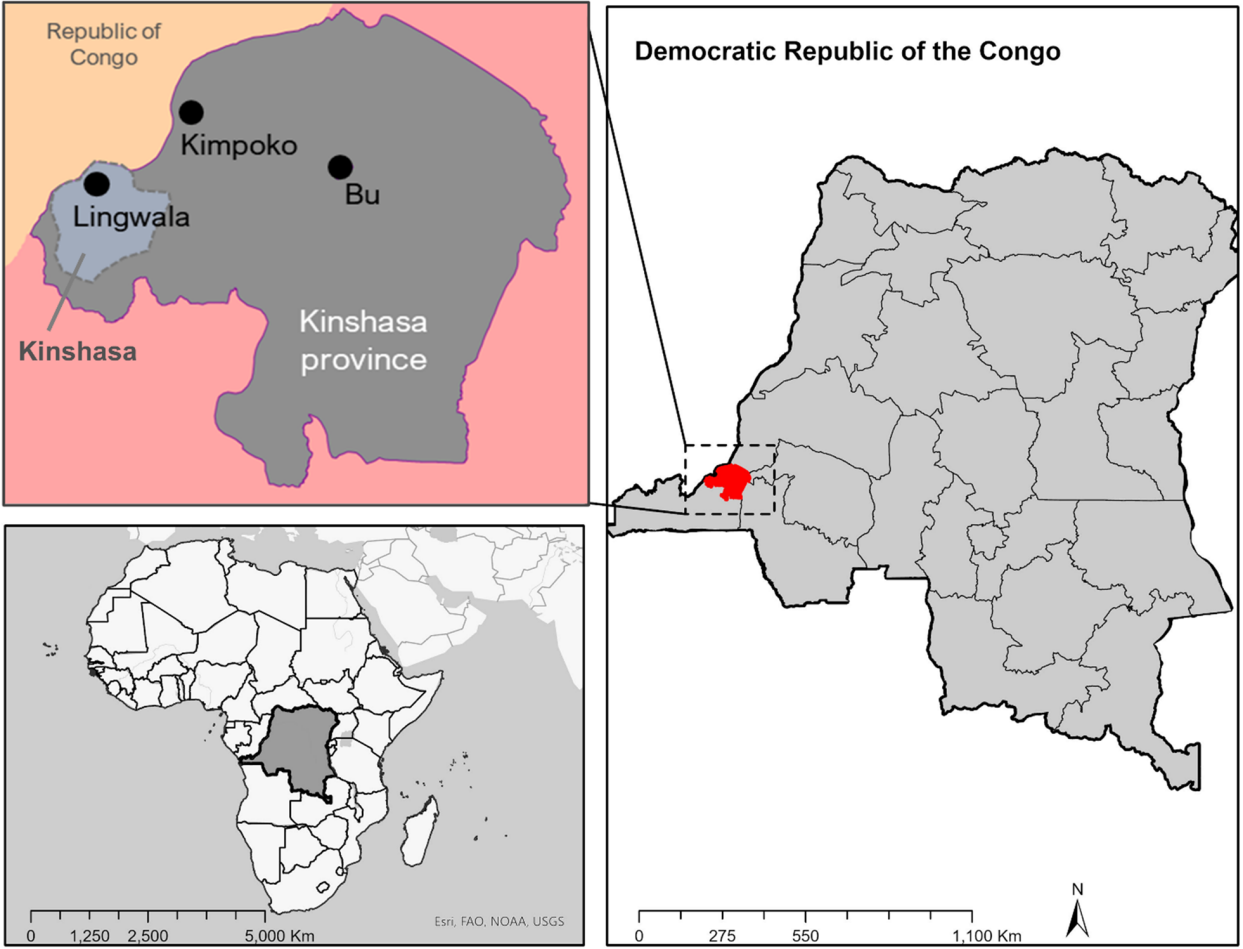
Internally generated map of study sites in Kinshsa Province, DRC

National staff members from the Kinshasa School of Public Health (KSPH) conducted primary data collection for this study in Kinshasa Province, DRC (see Fig. 1) in September of 2021. Qualifying by their respective levels of education, training, and field experience required to effectively perform the malaria RDT, teachers, CHWs, nurses, and laboratory technicians were recruited to participate across each core data collection phase—observation checklist assessment, surveys, and FGDs—at three distinct community sites in Kinshasa: Bû (rural), Kimpoko (semi-urban), and Lingwala (urban). Approximately 50 participants from each study site were recruited. Participants were purposively selected by KSPH community liaisons to represent expert and non-expert occupational groups who may be future end-users and key stakeholders of the SMAART-1 technology. The health professionals (e.g., CHWs, nurses, and laboratory technicians) represented expert users of the technology as they were sufficiently experienced with use of other RDTs, while teachers broadly represented non-expert users—individuals who have knowledge of and and experience with malaria and malaria testing in the communitythrough prior training, but may lack technical knowledge about specific elements of the technology. The overarching group of participants (*n* = 158) from the three sites that were trained in the use of the SMAART-1 protocol served as the sampling frame for the elicitation of inclusion in the survey and FGDs.

### Data collection

Three data collection methods were utilized in this study to provide an overarching acceptability evaluation of the SMAART-1 at PON field sites: observation checklist assessments, surveys, and FGDs. All consent-affirming language, study instructions, and instrument questions were provided in either French or Lingala, whichever language was preferred by the participant. Pilot testing of the tools was conducted among the KSPH team, which allowed for refinement of phrasing to ensure effective translations in French and Lingala. Initial data collection was paper-based and subsequently entered into EpiInfo 7.

Observation checklist assessments involved the appraisal of individual participants (*n* = 158), whereby KSPH study monitors referenced a targeted evaluative checklist to guide their appraisals of participants’ use of a mock SMAART-1 test and two saliva collection methods: passive saliva collection into a cup and the SUPER-SAL saliva collection device. The participants observed were those that were trained on the SMAART-1 test across the study sites. The checklist for the study monitors mirrored the instructional pamphlet given to study participants, with each step representing an item on the checklist (Table 2). Mock tests were incorporated into the study as a commercial product was not available at the time of data collection. Participants trialed the saliva detection methods using a demonstration RDT based on the design of the SMAART-1 test, then were provided a cassette containing a positive control test strip to simulate a mock result. This procedure was thoroughly explained during the informed consent process, with particular emphasis that the “result” cassette was not indicative of participants’ infection status.

Additionally, an additive score was generated based on the responses to the observation checklist. Trained observers were asked to denote “yes” if participant effectively carried out the step in the protocol or “no” if they had not. An observed efficacy score was created by summing the responses to these questions. Based on the number of steps effectively carried out, a participant would receive an associated score, whereby the successful execution of a given single step resulted in a score of 1. A respondent who effectively completed each of the 21 steps in the protocol would therefore receive a cumulative score of 21.

KSPH enumerators conducted individual surveys following the observational assessment to record participants’ (*n* = 145) demographics and attitudes about the test and saliva collection methods. The survey consisted of nine Likert-type items related to three DOI constructs, one Likert-type question regarding participants’ perceived likelihood of adopting the SMAART-1 protocol, and standard demographic questions to collect data related to participants’ sex, age, education, occupation, and years of experience. The occupation was was originally coded as 0 to 5 (0 = Community health worker, 1 = Nurse, 2 = Doctor, 3 = School teacher, 4 = Laboratory technician and 5 = other). To create additional opportunities for analysis, the occupation variable was transformed into a new dichotomous variable that organized the respondents as “health professionals” that were coded as 1 and “non-health professionals” that were coded as 2 to understand if there were any relationships based on this re-classification of occupation types.

The Likert-type DOI-related items included in this inquiry are listed below:

- The saliva-based malaria asymptomatic rapid test fits well with our malaria detection and treatment programme (Compatibility)
- Using the saliva-based malaria asymptomatic rapid test will improve the ability to treat malaria quickly (Relative Advantage)
- The saliva-based malaria asymptomatic rapid test is better (i.e., facilitates an improved user experience and/or is more effective and accurate) than the malaria detection tests I have used in the past. (Relative Advantage)
- Overall, there are benefits to using the saliva-based malaria asymptomatic rapid test (Relative Advantage)
- The saliva-based malaria asymptomatic rapid test is complicated (Complexity)
- The saliva-based malaria asymptomatic rapid test would be difficult to use (Complexity)
- It would be easy for me to become skillful at using the saliva-based malaria asymptomatic rapid test (Complexity)
- It would be easy to test adults using the saliva-based malaria asymptomatic rapid test (Complexity)
- It would be easy to test children using the saliva-based malaria asymptomatic rapid test (Complexity)

Respondents were asked to rate their level of agreement with each item on a five-point Likert-type scale of agreement where 1 = strongly disagree and 5 = strongly agree. Items with negative connotations of the technology were reverse coded for analysis. The Cronbach’s Alpha score for these items was 0.74, denoting an acceptable reliability coefficient for a social science construct [18]. Responses for the nine DOI-related items were initially coded from 0 (*strongly disagree*) to 4 (*strongly agree*). An average value from the nine items was subsequently calculated for each individuals to create a Technology Perception Index (TPI) that signified the extent to which a respondent had a positive or negative perception of the technology. A mean TPI value approaching 0 therefore represented a highly negative perception of the technology while a mean TPI value approaching 4 represented a highly positive perception of the technology. In addition to the items that make up the TPI, there was an additional item that used the same likert-type agreement scale to understand if participants would use the technology, “*I would use the saliva-based malaria asymptomatic rapid test as part of a community malaria detection and treatment programme*.”

Finally, KSPH study facilitators conducted a total of six recorded FGDs engaging six to seven participants (split as evenly as possible according to gender, sex and age within each session) from each of two core participant segments under consideration: health professionals (including CHWs, nurses, and lab technicians) and highly educated and trained teachers with broad experience with malaria testing in their respective communities. These participants were selected from the individuals that were trained on and observed using the SMAART-1 protocol. Three focus group sessions were conducted with each participant group, and two sessions—one per occupational group—were conducted per study site (i.e., one FGD with CHWs and one FGD with teachers for each target community: Bû, Kimpoko, and Lingwala). Each of the six FGDs was informed by a semi-structured topic guide focusing on participants’ previous experience with malaria detection and testing, perceptions of new saliva-based technology, and considerations for use and broader adoption of the SMAART-1 protocol. The KSPH study facilitators generated direct-language transcriptions and English translations of each FGD, removing any names or identifying information in the process to ensure participant anonymity and confidentiality. These transcript files (as well as original audio files) were uploaded to a secure (i.e., encrypted) remote-sharing folder only accessible to study staff.

### Data analysis

Observation checklist assessment data were analuzed using IBM SPSS Statistics version 26.0 primarily applying descriptive statistics to examine the frequencies/percentages of participants who were able to effectively carry out each step of the protocol. Relatedly, measures of central tendency for participants’ observation efficacy scores were determined in terms of median, range, mode and interquartile range. Nonparameteric correlations were conducted using Pearson correlation coefficient including the efficacy score and demographic variables to understand if there were relationships in scores based on age, education, occupation and years of professional experience. Relatedly, efficacy scores based on age were analysed using descriptive statistics, as a result of the dichotomous nature of the variable, and presented qualitatively. Though the core analysis of checklist data was conducted quantitatively (i.e., using statistics), notations generated by protocol checklist observers/moderators provided textual data that was incorporated into the qualitive analysis of FGDs discussed below.

For the survey data, descriptive statistics were applied to calculate frequencies/percentages of participants per demographic characteristics. Descriptive statistics were also used to determine the mean and standard deviation of the likert-type items and the TPI. Multiple Pearson non-parametric correlations were run to identify potential relationship between the TPI, the efficacy score, agreement to use the technology variable, and demographic characteristics. Table 1 outlines the correlation cefficiencts strength interpretations used in this study and informed by Schober et al. [19]. The following stratifications were applied to interpret the strength of the correlations based on the correlation coefficiencts: Since sex was a dichotomous variable, it was excluded from correlational analysis and the mean and standard deviation of the TPI and agreement to use the technology variable by sex was instead provided.

**Table 1:** Correlation coefficient interpretations

Correlation coefficient	Interpretation
0.00–0.10	Negligible correlation
0.10–0.39	Weak correlation
0.40–0.69	Moderate correlation
0.70–0.89	Strong correlation
0.90–1.00	Very strong correlation

Analysing FGDs first required the translated transcriptions of both Lingala and French-based session recordings into English—a process facilitated by KSPH staff members fluent in each language. Translated transcription files were accessed from a secure shared folder, whereby a qualitative analyst on the study team uploaded the files into the qualitative data analysis software program NVivo (Version 12). Transcript files were analysed using thematic analysis, whereby emergent categories and themes were inductively generated from the data using open coding [20]. After applying the initial step of fragmenting data into conceptual components to develop primary thematic categories, the process of identifying consistent patterns and relationships between disparate segments of data produced sub-themes, or “child” codes, nested within the first tier—or “parent”—conceptual categories [20]. Examples of both primary categories and nested codes are provided in the results section. The development and aggregation of categories and codes was determined to be complete once the lead analyst felt data saturation (i.e., the point in analysis where no new themes emerge) was attained [20].

To meet qualitative evaluation criteria identified by Lincoln and Guba [21], select techniques were applied to verify the credibility (confidence in the “truth” of findings) and confirmability (a measure of analyst neutrality or bias) of the preliminary analysis. The lead analyst exported and modified a codebook from NVivo into an Excel spreadsheet to share analysis results with other members of the study team. Adhering to recommendations from Bernard et al. [20] and Ryan and Bernard [22], the codebook included brief descriptions of thematic categories, inclusion and exclusion criteria (i.e., brief notes detailing what constituted a given category or code), and data exemplars for each code (i.e., representative quotes). To improve the credibility of preliminary findings, analyst triangulation was employed [22]. The initially developed codebook contained columns for additional research team members to provide feedback regarding theme appropriateness, structure, or phrasing. The codebook file also contained notations contextualizing and clarifying the primary analyst’s rationale for theme identification and organization—a reflexive peer debriefing exercise implemented to address potential confirmability bias concerns [23]. Once all eligible study team members reviewed and provided their comments to codebook file, the lead FGD analyst incorporated all revisions, adopting suggested changes to theme structure, phrasing, and placement.

## Results

### Participant socio-demographic characteristics

Table 2 outlines the socio-demographic characteristics of participants involved in both the survey and observation checklist data collection components of this study. Though specific demographic data were not collected from individuals involved in the FGDs, those individuals were drawn from this overarching sampling frame, and are therefore represented within the demographic characteristics presented in Table 2. In the same table, there is the designation of “other” in the occupation section of demographics. Of those seven individuals, there was an officer/official, a pastor, a supervisor, someone in charge of school sanitation, a fisherman, someone in charge of surveillance, and a saleswoman.

**Table 2:** Socio-demographic characteristics of observation checklist and survey participants

Participant characteristics (N = 139)	%	(n)
Sex		
Male	72.3	102
Female	27.7	39
Age		
18–24	3.6	5
25–34	18.7	26
35–44	32.4	45
45–54	26.6	37
55–64	14.4	20
65–74	3.6	5
75+	0.7	1
Education		
Secondary	81.0	115
University	19.0	27
Occupation		
Community health worker	30.9	43
Nurse	12.9	18
Doctor	0.7	1
School teacher	48.2	67
Laboratory technician	2.2	3
Other	5.0	7
Years of professional experience		
Less than 1 year	2.2	3
1–5 years	19.4	27
6–10 years	29.5	41
11–14 years	15.8	22
15 or more years	33.1	46

### Observation checklist assessment results

Table 3 outlines the numbers and percentages of participants who were able to effectively carry out each step of the SMAART-1 protocol. Overall, the participants observed efficacy scores that ranged from 5 to 21, with a mode of 16. The participant median score was 21 with an interquartile range of 1. Pearson correlation coefficients were computed to assess the linear relationships between the efficacy scores and demographic characteristics (except for sex) of the respondents. There were two significant relationship that existed. There was a weak, positive correlation between the efficacy score and age, r (125) = 0.180, *p* = 0.043. There was also a weak, positive correlation between efficacy score and years of professional experience, r (125) = 0.201, *p* = 0.024. This means that there is a weak relationship with older respondents with more professional years of experience and higher efficacy scores. With the original coding of the occupation variable, there was not a significant relationship between occupation and efficacy but the recoded variable is used, a significant relationship is identified. There is a weak, negative relationship between recoded occupation and efficacy scores, r (130) =  − 0.272, *p* = 0.002. This means that the health professionals scored marginally higher efficacy scores than the non-health professionals, which is not surprising. When looking at sex, females and males exhibited the same median (21) and iterquartile range (1) as the entire sample.

**Table 3:** Summary table of participant observation data for SMAART-1 technology test

Observational checklist	No % (*n*)	Yes % (*n*)
Placed contents on a clean and dry surface	4.1 (6)	95.9 (140)
Leaned forward at a 45-degree angle	14.5 (21)	85.5 (124)
Aligned medicine cup below mouth	5.5 (8)	94.5 (137)
Allowed saliva to collect in medicine cup	3.4 (5)	96.6 (140)
Collected at least 100 µL of saliva in medicine cup	2.7 (4)	97.3 (142)
Observed that subject did not eat or drink during collection	2.1 (3)	97.9 (142)
Took up 100 µL of saliva from medicine cup into transfer pipette	3.4 (5)	96.6 (141)
Aligned transfer pipette over sample well of cassette	2.7 (4)	97.3 (142)
Transferred 100 µL of saliva onto sample well of cassette	2.7 (4)	97.3 (142)
Placed contents on a clean and dry surface	1.4 (2)	98.6 (140)
Placed the tip end of the white absorbent collection pad of the Super-SAL device into the mouth, where saliva pools	7.0 (10)	93.0 (132)
Observed that subject did not chew or suck on absorbent collection pad during sample collection	4.9 (7)	95.1 (135)
Observed that subject did not eat or drink during collection	.70 (1)	99.3 (141)
Collected saliva until the pad was saturated	10.6 (15)	89.4 (127)
Placed the white absorbent pad end into the Plastic Compression Tube holding the Super-SAL device in an upright and vertical position	11.3 (16)	88.7 (126)
Aligned Plastic Compression Tube holding the Super-SAL device over sample well of cassette	5.6 (8)	94.4 (134
Pushed the plunger downward firmly to transfer saliva from the absorbent pad into the sample well of the cassette	7.1 (10)	92.9 (131)
Held plunger down for 15 s to ensure full transfer of saliva	8.5 (12)	91.5 (129
Placed UV-LED flashlight onto cassette	6.3 (9)	93.8 (135)
Illuminated test strip with UV-LED flashlight	8.4 (12)	91.6 (131)
Correctly recorded test outcome (positive, negative, inconclusive)	17.7 (25)	82.3 (116)

### Survey results

Overall, approximately 99% of the participants surveyed indicated that they either “agreed” or “strongly agreed” with the statement that they “would use the saliva-based malaria asymptomatic rapid test as part of a community malaria detection and treatment programme.” Only one individual in the sample of 144 (0.689%) indicated that they “disagreed” with the statement. Table 3 below provides univariate descriptive statistics for the likert-type items and the TPI.

When correlational analysis of the TPI, the efficacy score, demographic characteristics and the dependent variable was executed, multiple significant relationships were found. There was a weak positive correlation between the dependent variable and occupation, r (138) = 0.204, *p* = 0.017. When using the transformed occupation variable, the correlation coefficient increased but the relationship remained weak. There was a weak positive correlation between the dependent variable and the recoded occupation variable, r (138) = 0.217, *p* = 0.003. So, those that were not classified as health professionals rated there agreement higher than health professionals. There was strong correlation between the dependent variable and the TPI, r (144) = 0.740, *p* < 0.001. This means the more positive the perceptions of the technology based on the tenets of the DOI theory, the more someone agreed to use the saliva-based system in their community malaria detection and treatment programme. Relatedly, there was a weak positive correlation between occupation and TPI, r (137) = 278, *p* = 0.001. Meaning, those higher coded occupations (teachers, lab technicians, and others) had somewhat of a higher TPI than the CHWs, nurses, and doctors. When using the transformed occupation variable, the correlation coefficient again increased but the relationship remained week. There was a weak positive correlation between occupation and TPI, r (137) = 0.326, *p* < 0.001. This means that non-health professionals rated the technology more favourably in the TPI than the health professionals.

Because the TPI exhibited a strong relationship with the agreement to use SMAART-1 in community malaria detection and management programs, additional Pearson non-parametric correlations were executed between the individuals items of the TPI and the dependent variable. All but two items (item 6 and item 10; Table 4) were significant, which resided within the tenet of complexity within the DOI theory. Below is a list of the relationships that existed (Table 4 for item numbers):

- There was a moderate, positive correlation between the dependent variable and item 2, r (145) = 0.628, *p* < 0.001.
- There was a moderate, positive correlation between the dependent variable and item 3, r (145) = 0.622, *p* < 0.001.
- There was a moderate, positive correlation between the dependent variable and item 4, r (145) = 0.588, *p* < 0.001.
- There was a moderate, positive correlation between the dependent variable and item 5, r (145) = 0.617, *p* < 0.001.
- There was a moderate, positive correlation between the dependent variable and item 7, r (145) = 0.485, *p* < 0.001.
- There was a moderate, positive correlation between the dependent variable and item 8, r (145) = 0.621, *p* < 0.001.
- There was a moderate, positive correlation between the dependent variable and item 9, r (145) = 0.655, *p* < 0.001.

**Table 4:** Summary table of descriptive statistics of likert-type items

#	Item	Mean	Standard deviation
1	I would use the saliva-based malaria asymptomatic rapid test as part of a community malaria detection and treatment program (dependent variable)	3.62	0.541
2	The saliva-based malaria asymptomatic rapid test fits well with our malaria detection and treatment program	3.68	0.484
3	Using the saliva-based malaria asymptomatic rapid test will improve the ability to treat malaria quickly	3.67	0.487
4	The saliva-based malaria asymptomatic rapid test is better than the malaria detection tests I have used in the past	3.53	0.590
5	Overall, there are benefits to using the saliva-based malaria asymptomatic rapid test	3.67	0.472
6	The saliva-based malaria asymptomatic rapid test is complicated (reverse coded)	3.46	0.773
7	The saliva-based malaria asymptomatic rapid test would be difficult to use	3.27	1.029
8	It would be easy for me to become skillful at using the saliva-based malaria asymptomatic rapid test	3.61	0.719
9	It would be easy to test adults using the saliva-based malaria asymptomatic rapid test	3.61	0.490
10	It would be easy to test children using the saliva-based malaria asymptomatic rapid test	3.19	0.844
11	TPI	3.52	0.384

This demonstrates that the higher agreement ratings for perceived fit, relative advantage, and three out of the five complexity items are related to higher overall perceptions of the technology, which demonstrates higher agreement to use the SMAART-1 in community malaria detection and management programmes.

### Focus group results

The inductive (i.e., data-driven) analysis of FGDs—coupled with analysis of select observation checklist notations—ultimately generated five first-tier thematic categories: “Disadvantages of Blood RDT,” “Advantages of Saliva RDT,” “Disadvantages of Saliva RDT,” “Overarching Testing Considerations,” and “Diffusion Considerations”. These thematic (or conceptual) labels served to cluster and thematically organize broadly related segments of data, encompassing more specific themes (i.e., codes) within them. The first category—“Disadvantages of Blood RDT”—encompassed participants’ considerations towards traditional blood-based rapid diagnostic methods for assessing the presence of malaria infection. Five discrete codes were generated from our thematic analysis of participant responses within this category. These codes are represented in Table 5, with an associated datum exemplar (i.e., a representative quote) for each code.

**Table 5:** Disadvantages of blood RDT codes

Disadvantages of blood RDT codes	Representative quote
Detection inaccuracies (false negatives/high detection threshold)	The test was good, but you will see that even if someone is sick the test can be negative or if the person has malaria you have to go deeper by doing a thick blood smear to see that malaria is present, so we the medical professionals we believe that even if the RDT is negative, we still administer the malaria medicine as far as we have some clinical evidence
Inefficient/slow results	I would like to add this: this examination, as (the director) has just said, if blood is taken, the test result is not given on the spot; you will see that if you are tested in the morning, you have to come back in the afternoon
Supply/availability concerns	We have experienced enormous difficulties with the renewal of stocks/supplies, absence at the market level despite the availability of money. The moment the need arises, the Central Office of the Health Zone or the partners will make you wait (for the RDT)
Uncomfortable/painful	(The RDT) requires the (finger) prick and that's what's painful, especially for children. This pain leads some people to avoid malaria RDT testing
Unsanitary/contamination risk	The needle or blood is also dangerous for the caregivers with the risk of self-contamination

Teacher and health professional (including CHW, nurse, and lab technician) respondents from each community site that were previously trained and observed were subsequently prompted to discuss their perspectives—based on prior engagement and experience—of the use of saliva-based RDTs. In assessing the accessibility and overall efficacy of saliva-based testing protocols, participants provided a wide array of feedback, ultimately generating two overarching thematic categories: “Advantages of Saliva RDT,” and “Disadvantages of Saliva RDT”. “Advantages of Saliva RDT”—illustrated in Table 6 below—was further disaggregated into five unique codes. These codes are presented below in conjunction with associated representative quotes.

**Table 6:** Advantages of saliva RDT codes

Advantages of saliva RDT codes	Representative quote
Accurate detections from non-symptomatic people/small samples/early-stage detection	The saliva-based test is better or preferable because here the detection is much more sensitive, even if the microbe is only in its early stages
Ease of use	The technique was easy, very easy, they demonstrated, they first read the instructions, we showed them the demonstration, we asked them questions, it was really very easy
Efficient/quick results	But the saliva one, he (the patient) will just give the saliva and quickly he (the patient) will gets the result
Not painful/less intimidating than needle pricks	You will see someone who was pale, but you (can) take the saliva from them. For the blood (RDT) with another child it can turn into a fight, and this can bring an incident. You can have glassware break. Whereas with saliva (RDT), you can ask to spit, put the saliva in the jar/cup, you will see that it is fine
Will become more widely available/accessible	The saliva-based RDT can be done in all circumstances and by everyone wherever they are: at home, at the hospital, at school, at church, etc., provided that the test is available. For us it is a referral test for care. This means that I perform the test wherever I am, if it is positive, I bring the results to the health professional for treatment

Table 7 illustrates codes with the “Disadvantages of Saliva RDT” thematic category. Six discrete codes were distilled from FGD data within this category. Two additional codes, denoted by asterisks, were primarily identified by participant observation checklist notations—the textual feedback provided by checklist monitors in a preceding data collection phase. Each of these eight total codes is supported by a representative quote.

**Table 7:** Disadvantages of saliva RDT codes

Disadvantages of saliva RDT codes	Representative quote
Concerns that devices may be recycled/unsanitary	There is a risk that people will believe that the devices used are recycled. To circumvent this belief or thought, we suggest that the device be covered in a package that is only uncovered when used in front of the care recipient, like with syringes. Otherwise, people will avoid using these
Difficult to use with sick/inhibited/convulsing patients	There will be a small difficulty for people who have a seizure especially if they have convulsions or if the sample has already been taken for analysis as is generally required
Similarity to COVID-19 protocols (testing hesitancy)	(A) strong difficulty is related to COVID-19—a lot of things were said. People have developed a lot of resistance, so a lot of awareness is needed, because many things are said (about COVID-19)
Supply/availability concerns	Disadvantage will be that the test will not be available because many people will come to the Center, if there is a break (in supply) it is not good. So, avoid breakage (stockout) when this work will start
Difficult to stimulate/use saliva	It's a bit tricky for people who come in and don't have saliva, maybe they're given water beforehand?
Difficult to administer to elders (positioning, posture, etc.)^a^	RDT a little difficult for the older ones; difficulty in taking the ideal position (elbow on the knees)
Difficult to interpret results^a^	Difficulty in distinguishing the invalid (results) from the negative (results)

^a^Code generated from observation checklist assessment notation

Focus group participants were prompted to consider additional factors in diagnostic testing service, adoption, testing execution, etc. “Overall Testing Considerations” and its six constituent codes are presented in Table 8. In analysing data within this thematic category, two sub-themes were distilled from the “Design/Protocol Modification Suggestions” code identified. These sub-themes reflect an additional layer of granularity and nuance offered by participants. In the table below, the two sub-themes are denoted by an obelus (i.e., a dagger) mark.

**Table 8:** Overall testing consideration codes

Overall testing consideration codes	Representative quote
Need for promotion/expanded awareness	So this test is a new thing in the field, if you come to the Center and we say give the saliva, someone will not be afraid, we will explain to people, we do an awareness campaign because it is something new that comes to the field
Design/protocol modification suggestions	Should explain from time to time see that the 'circle' turns red; too much light disturbs the reading
Adapting devices for children^a^	It's good, easy, more direct; but for the children we must make the small size (saliva) device—this one is too big
Appropriate discard/waste management practices^a^	There is a risk that people will believe that the (saliva) devices used are recycled. To circumvent this belief or thought, we suggest that the device be covered in a package that is only uncovered when used in front of the care recipient, like with syringes. Otherwise, people will avoid using these
Expand availability	For me, the saliva-based malaria test must be integrated into the health program as well as into the health system. That is to say, it must be made available to everyone so that it can be done everywhere and by everyone, and that anyone who is positive is directed to the health center for appropriate care
Spitting cup/super sal device preference	It's easy, you spit in the cup, directly the technician puts the saliva in the cassette, easy; then you throw away the cup. The (saliva/SUPER-SAL) device takes time, I tried it myself, the mouth was dry, the saliva came with difficulty. Meanwhile you can catch germs when you put it back into the compression tube

^a^Sub-theme for “Design/Protocol Modification Suggestions” code

Finally, FGD participants provided critical feedback regarding diffusion-related considerations—factors related to the communication, promotion, and public sensitization of saliva RDTs, including which media channels and stakeholder groups are best-suited to advance SMAART-1 adoption. Table 9 illustrates this final “Diffusion” category and the four codes it encompasses. As in preceding tables, each code is supported by a representative quote.

**Table 9:** Diffusion codes

Diffusion codes	Representative quote
Spreading knowledge/awareness	We should organize open days (i.e., open house) where it will be used so that the community is aware of it, awareness campaigns, free tests on saliva and blood as we are doing; the detection should really be done in the eyes of the community, the news will spread everywhere
Communication/sensitization channels: CHWs/relays	It is the work of community health workers—if something is newly introduced, we will sensitize the population and show its advantage for children; to draw blood from them is a problem, we must sensitize the mothers, the adults, so that they easily understand that the thing we brought is like this, that even if malaria is in its early stage it is easily detected so it will be more advantageous
Communication/sensitization channels: teachers	In short: for us teachers, we are always available for activities related to health, but it is you who put us aside. Today you have looked for us, you see how we have come. We are ready to collaborate with you, especially if you go through the IT channel, because we promise you our support for the popularization of this saliva-based test, which we see is one of the best RDTs for malaria
Communication/sensitization channels: general media	Another brother adds the churches, the press, the media, he cites the radio…

## Discussion

The recognition of pervasive diagnostic capacity limitations of established malaria RDTs at PON and POC sites in malaria endemic regions—particularly throughout sub-Saharan Africa—presented a significant need to develop a diagnostic technology and testing protocol sufficiently sensitive to low-density/subclinical *P. falciparum* infection [12]. This need has been deemed more critical based on assertions that frequent failures of existing diagnostic test methods (including microscopy and blood-based RDTs) to accurately and consistently detect subclinical infection in high-risk regions have likely obstructed global malaria elimination progress over recent years [10, 12]. The collaborative development of the SMAART-1 protocol offers an opportunity to close the subclinical detection gap, with clinically reliable results demonstrating a promising new level of sensitivity and precision for detecting parasite bio-markers. While clinical results were promising, evaluating the protocol’s utility and adoption potential in the field, with a target user audience—the primary objective of this study—became paramount to advance its development.

In this study, the adoption potential of the SMAART-1 was appraised through three data collection methods at target PON sites in Kinshasa Province, DRC: surveys, observation checklist assessments, and FGDs. Observational checklist data—whereby trained teacher and health professional participants’ (*n* = 158) adoption of a mock SMAART-1 protocol and two saliva collection methods was assessed by KSPH study monitors—indicate that participants were generally able to administer the SMAART-1 without significant obstacles. With a median efficacy score of 21 and an interquartile range of 1, checklist results demonstrate a relatively uniform proficiency for use across our participant types. Because only weak relationships existed between the efficacy scores and a participants occupation and years of professional experience, this reinforces the above statement and demonstrates the relative ease of use across experts and non-experts.

Survey findings also indicate that perceptions of the SMAART-1 play a significant role in community members’ likelihood to adopt the protocol. The TPI, developed based on tenets of the DOI theory, demonstrated a strong positive relationship with the agreement to use the saliva-based system in malaria detection and management programs. While both relative advantage and complexity were significant, there seems to be more relationships and opportunities to frame messages and education centered on the relative advantage of SMAART-1 versus previous options for malaria detection. Relatedly, our findings that occupation (considering all distinct roles within the two overarching occupational categories of teachers and health professionals) demonstrates a weak relationship not only with actual use of the technology but also intended use and technology perceptions (i.e., a TPI score) suggests that there may exist opportunities for health professionals and non-health professionals alike to champion and administer the technology with comparable efficacy. According to DOI theory, these extant perceptions can be influenced by targeted sensitization and educational efforts implemented to familiarize potential users with a target innovation and reduce uncertainty [14]. Following the contention within DOI theory that opinion leaders are instrumental in the diffusion of new technologies, ideas, and behaviors through a target population, malaria test technology “influencers” (e.g., teachers and health professionals) could be identified based on their experience and occupational expertise to promote SMAART-1 to peer-group members across malaria-endemic region and communities comparable to those featured in this study [14]. This recommendation has precedent in infectious disease treatment work broadly and malaria control work specifically. Marshall et al. [24] leveraged the DOI framework to identify the most salient structural factors that could facilitate or inhibit the widespread promotion and adoption of direct-acting anti-viral therapies for hepatitis C virus. The researchers similarly recognized that individuals with specialized occupational expertise comprised the most innovative and influential adopter and diffuser (i.e., promoter) of an innovation, finding that drug and alcohol specialists—rather than general practitioners—were most likely to aid the scaling up of anti-viral treatments [24]. In the malaria context, Steury [25] utilized the DOI to develop an applied model to implement community-focused malaria treatment invertions in Zambia. In this model, CHWs were similarly targeted for their experience and occupational expertise within a target Zambian community to introduce, deliver, and promote long-lasting ITNs to potential adopters in a systematic way (i.e., by targeting innovators and early-adopters such as the township chief and township elders) [25]. In this study context, there exists the opportunity to train both experts and non-experts alike broadening the opportunities for these influencers to mitigate misperceptions of “compatibility”, “relative advantage” and “complexity” of the SMAART-1.

FGDs with teachers and CHWs generated several salient findings—some previously corroborated in prior malaria prevention and treatment studies, others emergent and seemingly novel to this study. One salient theme that emerged from the FGDs was that SMAART-1 and other saliva-based RDTs carry a distinct advantage over conventional blood-based RDTs given they do not require finger pricks or the use of a needle. In their systematic review of qualitative studies examining malaria prevention and treatment barriers throughout Africa, Maslove et al. [26] found that the fear of needles and perceived adverse effects from injections (including death) were major barriers to communities’ likelihood of receiving blood-based malaria treatments. Similarly, a recent international survey of National Malaria Control Programme (NMCP) representatives demonstrated that the need and impact potential for non-invasive tests was significant, with suggestion that saliva-based test products that did not utilize needles or extract blood could be viewed as less invasive, and more likely to be readily adopted by test subjects—children and adults alike [27]. Additionally, findings indicate these less invasive (i.e., non blood-based) RDTs would generally require minimal training and expertise to administer, increasing its widespread adoption potential [27]. These and other results suggest that the non-presence of needles/non-requirement of injections in a diagnostic testing protocol may alleviate fears and facilitate a greater likelihood to seek and receive treatment.

Another recurring theme concerning blood-based RDT disadvantages was primarily identified by lab technicians within the health professional FGDs and related to detection inaccuracies—namely the frequency of false negatives due to blood-based RDT’s extremely limited detection capacities when assessing asymptomatic or subclinical infections. According to Owusu et al. [27], NMCP representatives identified both RDT product quality and stability as major global malaria control challenge, with many survey respondents stating a preference for “…saliva-based malaria RDTs that could detect sub-microscopic infections and could be used by untrained lay people” [27]. This suggests growing recognition of the need for innovative, yet accessible saliva-based RDT products with the requisite clinical sensitity to accurately detect low-density asympotamic infections.

Several other FGD themes emerged as salient considerations for health care practitioners in Kinshasa province, including opportunities for modifying elements of the SMAART-1 protocol to improve its use for children, recognizing potential issues with administering the test with unconscious, convulsing, or otherwise infirm individuals, promoting the protocol based on recognized accessibility and ease of use, and leveraging the expertise and trusted status of teachers, CHWs, and other key stakeholders in these target communities to help promote SMAART-1 and sensitize community members to the ease, utility, and importance of taking the test—both as a preventative measure and to ensure effective treatment. These and other themes presented in this study warrant further exploration in comparable study settings.

### Study limitations

Limitations are present in this study. One previously addressed limitation pertains to the truncated operationalization of Roger’s DOI framework [14]. While the decision to abbreviate examination of all five DOI characteristics was made—in consulation with local partners and enumerators—on legitimate and valid grounds (i.e., with consideration for respondent survey fatigue and time availability), the exclusion of trialability and observatility items in the survey prevented the opportunity to examine additional relationships between these index items and participants’ likelihood to adopt. Given FGD data demonstrated participants were motivated by both the prospect of observing their peers’ use of the SMAART-1 and trialing the protocol for community members to promote its adoption, trialability and observability appear to be potentially salient constructs that warrant more explicit integration into future data collection efforts.

## Conclusions

In recent years, the achievability of key 2030 malaria elimination and mitigation goals set by the WHO have been called into question, prompted by the observed persistence—and in some regions, documented increases—of malaria infection and mortality. In response to concerns that subclinical infections may be a salient contributing factor to this stalling out of progress, a cross-sectoral partnership produced a saliva-based RDT sensitive and precise enough to successfully detect subclinical infections. While SMAART-1 proved clinically successful, its utility at PON and POC community sites in high-risk, malaria endemic regions was indeterminate. The purpose of this study, therefore, was to assess the acceptabilityand adoption potential of the SMAART-1 at targeted community sites in Kinshasa province, DRC through observation checklist assessments of SMAART-1 implementation, FGDs, and surveys with local health care practitioners. Primary findings indicate participants were interested in and supportive of the SMAART-1 protocol, with approximately 99% of the participants surveyed indicating that they either “agreed” or “strongly agreed” with the statement that they “would use the saliva-based malaria asymptomatic rapid test as part of a community malaria detection and treatment program.” Additional findings suggest that while the protocol was broadly appealing for its testing sensitivity and ease of use, a suite of opportunities remain to be pursued, including: addressing perceptional biases through community-based communication and sensitization campaigns, considering targeted design modifications for children and impaired individuals, and expanding research-based evaluation efforts in different countries and/or endemic regions to gauge the generalizability of the present study’s results.

## Data Availability

The instruments, datasets, and outputs generated for this study are available from the corresponding author upon reasonable request.

## Abbreviations

CHWs: Community health workers
DOI: Diffusion of innovation
DRC: Democratic Republic of the Congo
FGDs: Focus group discussion
IRS: Indoor residual spraying
ITNs: Insecticide-treated nets
KSPH: Kinshasa School of Public Health
NMCP: National Malaria Control Programme
POC: Point-of-care
PON: Point-of-need
RDT: Rapid diagnostic test
SMAART-1: Saliva-Based Malaria Asymptomatic and Asexual Rapid Test
TPI: Technology Perception Index
WHO: World Health Organization
